# Supplementary material for: Modeling glioblastoma heterogeneity as a dynamic network of cell states
Source: Mol Syst Biol. 2021 Sep 16;17(9):e10105. doi: 10.15252/msb.202010105 (PMC8444284; doi:10.15252/msb.202010105)
Supplement: Supplementary file 6 — Source Data for Figure 5 [file MSB-17-e10105-s004.zip › Figure5A_sourcedata/GSEA_3017/nosigdb_stateE.GseaPreranked.1622795031093/IVY_CTPAN.html]

Details for gene set IVY\_CTPAN[GSEA]

|  || Dataset | state63017 |
| Phenotype | NoPhenotypeAvailable |
| Upregulated in class | na\_pos |
| GeneSet | IVY\_CTPAN |
| Enrichment Score (ES) | 0.6886662 |
| Normalized Enrichment Score (NES) | 3.4735951 |
| Nominal p-value | 0.0 |
| FDR q-value | 0.0 |
| FWER p-Value | 0.0 |
Table: GSEA Results Summary

  

Fig 1: Enrichment plot: IVY\_CTPAN      
 Profile of the Running ES Score & Positions of GeneSet Members on the Rank Ordered List

  

| PROBE | GENE SYMBOL | GENE\_TITLE | RANK IN GENE LIST | RANK METRIC SCORE | RUNNING ES | CORE ENRICHMENT || 1 | CYR61 |  |  | 3 | 1.169 | 0.0950 | Yes |
| 2 | IGFBP5 |  |  | 4 | 1.015 | 0.1802 | Yes |
| 3 | CD44 |  |  | 14 | 0.806 | 0.2381 | Yes |
| 4 | CEBPD |  |  | 15 | 0.791 | 0.3046 | Yes |
| 5 | LGALS3 |  |  | 25 | 0.697 | 0.3534 | Yes |
| 6 | EMP1 |  |  | 40 | 0.584 | 0.3873 | Yes |
| 7 | S100A10 |  |  | 51 | 0.527 | 0.4207 | Yes |
| 8 | SERPINE1 |  |  | 52 | 0.526 | 0.4648 | Yes |
| 9 | PTX3 |  |  | 60 | 0.496 | 0.4989 | Yes |
| 10 | PDPN |  |  | 79 | 0.438 | 0.5162 | Yes |
| 11 | DOK5 |  |  | 81 | 0.437 | 0.5518 | Yes |
| 12 | SYTL2 |  |  | 82 | 0.437 | 0.5885 | Yes |
| 13 | IRS2 |  |  | 85 | 0.426 | 0.6222 | Yes |
| 14 | ATF3 |  |  | 101 | 0.397 | 0.6393 | Yes |
| 15 | IGFBP2 |  |  | 102 | 0.395 | 0.6724 | Yes |
| 16 | SOCS3 |  |  | 141 | 0.342 | 0.6600 | Yes |
| 17 | BNIP3L |  |  | 142 | 0.341 | 0.6887 | Yes |
| 18 | JUN |  |  | 172 | 0.316 | 0.6838 | No |
| 19 | SPP1 |  |  | 261 | 0.257 | 0.6101 | No |
| 20 | TGIF1 |  |  | 318 | -0.263 | 0.5715 | No |
| 21 | CA12 |  |  | 325 | -0.263 | 0.5871 | No |
| 22 | ARL4C |  |  | 453 | -0.300 | 0.4747 | No |
| 23 | SNHG1 |  |  | 538 | -0.328 | 0.4113 | No |
| 24 | EIF4EBP1 |  |  | 592 | -0.353 | 0.3835 | No |
Table: GSEA details [plain text format]

  

Fig 2: IVY\_CTPAN: Random ES distribution      
 Gene set null distribution of ES for **IVY\_CTPAN**
